# Integrin expression profiling identifies integrin alpha5 and beta1 as prognostic factors in early stage non-small cell lung cancer

**DOI:** 10.1186/1476-4598-9-152

**Published:** 2010-06-17

**Authors:** Anne-Marie C Dingemans, Vivian van den Boogaart, Bettine A Vosse, Robert-Jan van Suylen, Arjan W Griffioen, Victor L Thijssen

**Affiliations:** 1 Department of Pulmonology, Maastricht University Medical Center, P. Debyeplein 25, Maastricht, 6229 HX, The Netherlands; 2 Department of Pathology, Maastricht University Medical Center, P. Debyeplein 25, Maastricht, 6229 HX, The Netherlands; 3 Angiogenesis laboratory, Department of Medical Oncology, VU University medical center, De Boelelaan 1118, Amsterdam, 1081 HV, The Netherlands; 4 Angiogenesis laboratory, Department of Radiotherapy, VU University medical center, De Boelelaan 1118, Amsterdam, 1081 HV, The Netherlands

## Abstract

**Background:**

Selection of early stage non-small cell lung cancer patients with a high risk of recurrence is warranted in order to select patients who will benefit from adjuvant treatment strategies. We evaluated the prognostic value of integrin expression profiles in a retrospective study on frozen primary tumors of 68 patients with early stage non-small cell lung cancer.

**Methods:**

A retrospective study was performed on frozen primary tumors of 68 early stage non-small cell lung cancer patients with a follow up of at least 10 years. From all tumor tissues, RNA was isolated and reverse transcribed into cDNA. qPCR was used to generate mRNA expression profiles including integrins alpha1, 2, 3, 4, 5, 6, 7, 11, and V as well as integrins beta1, 3, 4, 5, 6, and 8.

**Results:**

The expression levels of integrins alpha5, beta1 and beta3 predicted overall survival and disease free survival in early stage NSCLC patients. There was no association between integrin expression and lymph node metastases. Comparison between the histological subtypes revealed a distinct integrin signature for squamous cell carcinoma while the profiles of adenocarcinoma and large cell carcinoma were largely the same.

**Conclusion:**

Integrin expression in NSCLC is important for the development and behavior of the tumor and influences the survival of the patient. Determining the integrin expression profile might serve as a tool in predicting the prognosis of individual patients.

## Introduction

Lung cancer is the most common cause of cancer related death in Europe, accounting for one-fifth of the total number of cancer deaths[1]. The prognosis of patients with non-small cell lung cancer (NSCLC), which constitutes 80% of all lung cancers, is poor, even in patients with early stage disease[2]. The five year survival rate of patients with resected NSCLC is between 50-60%, and 50-60% of patients will have disease recurrence within 2 years[3]. Recently, it has been shown that adjuvant chemotherapy improves overall and disease-free survival in patients with resected stage stage II-IIIa NSCLC, but not all patients benefit[3]. Thus, selection of patients with high risk of recurrence is warranted.

An important predictor of poor prognosis in patients with NSCLC is early distant metastasis[2]. When cancer cells become metastatic, they develop altered affinity and avidity for the extracellular matrix (ECM). This phenotypic change is partly due to alterations in the expression of cell-surface molecules known as integrins. Integrins are a family of glycoproteins that form heterodimeric receptors for ECM molecules such as laminin, fibronectin and collagen[4]. Each integrin generally consists of a non-covalently linked α- and β-subunit, with each subunit having a large extracellular domain, a single membrane-spanning domain, and a short, non-catalytic cytoplasmic tail[4]. Besides their role in mediating interactions of cells with the extracellular matrix, integrins also participate in signal transduction and influence different cell functions such as migration, differentiation, proliferation and apoptosis[5,6]. Consequently, integrins are involved in different steps of tumor progression[7].

The expression of integrins has been suggested to play a role in predicting the clinical course and prognosis of NSCLC[8-13]. For example, increased expression of integrin alpha5 and beta1 has been associated with lymph node metastasis. Increased expression of integrin alpha5 may also be a predictor of a poor 5-year survival rate[8,11]. However, integrin expression in NSCLC remains ambiguous[13]. The expression of integrin alpha3 has been found increased as well as decreased in adenocarcinoma of the lung and has been suggested a factor of poor prognosis[9,10]. Loss of integrin alphaV expression has been linked to recurrence in patients with lymph node-negative lung carcinoma, but high expression of this integrin has also been associated with poor patient outcome[12,14]. Apart from these conflicting findings, most studies only assess the expression of a single or a few integrins and information on the relation between the overall integrin expression profile and disease progression is scarce. To gain better insight in the prognostic value of integrin expression in NSCLC, we performed extensive integrin expression profiling in primary tumors of patients with resected early stage NSCLC.

## Patients and Methods

### Patients

Primary tumor tissue was obtained from patients with NSCLC who underwent a surgical resection between 1995 and 1999. Exclusion criteria were 1) previous other malignancy, or 2) development of an unrelated malignancy during a follow-up of at least 4 years. Both frozen tumor tissues (-80°C) and formalin fixed paraffin embedded tissues were obtained from the Maastricht Pathology Tissue Collection. Frozen tissue sections (8 μm) were stained with hematoxilin/eosin and re-evaluated by an experienced pathologist (R-J v S). Only tissues with a tumor area >50% were included for further investigations. The study complies with the recommendations guiding physicians in biomedical research involving human subjects as laid down in the Declaration of Helsinki.

### RNA isolation and cDNA synthesis

Total RNA was isolated from ten × 10 μm thick frozen tissue sections using RNeasy RNA isolation kit (Qiagen) according to the manufacturer's instructions. Possible genomic DNA contaminations were removed by on column DNAse treatment with the RNase-free DNAse set (Qiagen). cDNA synthesis was performed with the iScript cDNA Synthesis Kit (BioRad) using 100 ng total RNA as input.

### Primers design

Primers were designed as described previously[15]. Primers were targeted against integrin alpha1 (INTa1), alpha2 (INTa2), alpha3 (INTa3), alpha4 (INTa4), alpha5 (INTa5), alpha6 (INTa6), alpha7 (INTa7), alpha11 (INTa11), alphaV (INTaV), beta1 (INTb1), beta3 (INTb3), beta4 (INTb4), beta5 (INTb5), beta6 (INTb6), beta8 (INTb8), beta-actin (bACT), cyclophilin A (cycloA), beta-2-microglobulin (b2MG) and hypoxanthine-guanine phosphoribosyltransferase 1 (HPRT1). The primers specifically target human sequences and were synthesized by Eurogentec.

### Real-time qPCR

Real-time qPCR was performed on the iQ5 Multicolor Real-Time PCR Detection System device (BioRad) and the CFX96 (BioRad) using the iQ SYBR Green PCR master mix (BioRad). The PCR reaction was performed in a 25 μL volume containing 30 ng cDNA, 12.5 μL 2× iQ SYBR Green PCR master mix and 1 μL of primer mix (10 μM forward primer, 10 μM reverse primer). The PCR profile was as follows: 10 minutes at 95°C, followed by 40 cycles of 15 seconds at 95°C and 30 seconds at 60°C. Subsequently, a melting curve analysis was performed which consisted of 70 cycles of 10 seconds with a temperature increment of 0.5°C/cycle starting from 60°C. The obtained Ct value of each gene of interest was normalized to the Ct of the reference genes as follows: Ct_norm _= Ct_goi _- Ct_ref _with Ct_ref _= (Ct_bACT _× Ct_CycloA _× Ct_b2MG _× Ct_HPRT_)^(1/4) ^with _norm _= normalized, _goi _= gene of interest, and _ref _= reference gene.

### Statistical analysis

The real time RT-PCR data are given as mean values ± SEM. Survival comparisons were done using Kaplan-Meier survival estimates and the log-rank test for determining survival differences. Median integrin expression level was used as cut of value to divide the groups in high and low expression. The Student's *t *test was used to compare expression between the different histological groups. Statistical computations were performed in SPSS 12.0.1. and p-values < 0.05 were considered statistically significant.

## Results

### Patient characteristics

Of the initial 71 patients included in this study, one was excluded because of poor quality of the tissue and two were excluded because of incomplete follow-up. The average age of the remaining 68 patients was 69.1 years (range 44.5 - 90.2). None of the patients received adjuvant chemotherapy. The median follow-up was 93 months (95% CI 85 - 101 months) and the median overall survival was 27.3 months (range 2.3 - 126.7). During the follow-up period, 43 patients (67%) had disease recurrence and the 2 year survival rate was 51%. The patients characteristics are summarized in Table 1.

**Table 1:** Characteristics of included NSCLC patients.

**Total number of patients**	68
**Median age in years **(range)	69.1 (44.5 - 90.2)
**Sex**	
Male	51 (75%)
Female	17 (25%)
**Histology**	
Adenocarcinoma	24 (35%)
Squamous cell carcinoma	33 (49%)
Large cell carcinoma	10 (15%)
Pleiomorph carcinoma	1 (1%)
**Stage **(TNM)	
Stage IA	6 (9%)
Stage IB	34 (50%)
Stage IIA	6 (9%)
Stage IIB	18 (26%)
Stage IIIA	4 (6%)
**Smoking status**	
Never	2 (3%)
Former	42 (62%)
Current	19 (28%)
Unknown	5 (7%)
**Median OS in months **(range)	27.3 (1.7-126.7)
**Median DFS in months **(range)	16.6 (1.6 - 125.2)

### Integrin primer design and validation

**Figure 1:**
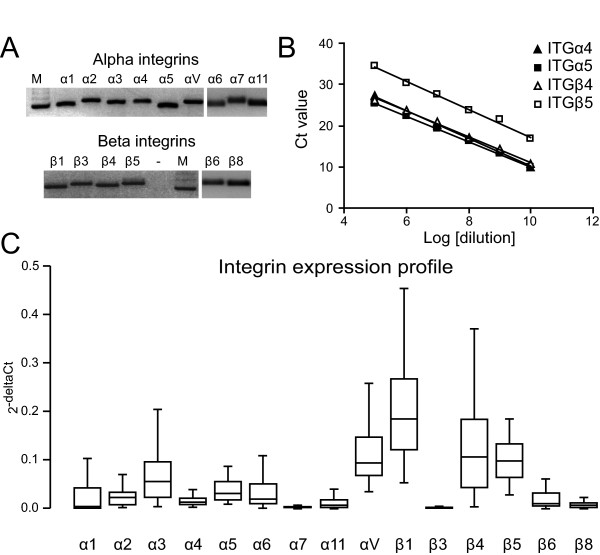
**Integrin expression profile in NSCLC**. **A) **Agarose gel electrophoresis of the specific integrin fragments. M = 100 bp DNA marker. **B) **Representative dilution curves of 4 integrins showing a broad linear range of detection. Ct = Cycle threshold. **C) **Box plot of the overall integrin expression profile in NSCLC tissues.

A subset of 15 integrins was selected covering the binding to different ligands, i.e. collagen, fibronectin, laminin, vitronectin, and von Willebrand's factor. Primers were designed to specifically target only human integrins as described previously[15] (Table 2). Specific detection of a particular integrin was confirmed by agarose gel electrophoresis (Figure 1A). To ensure equal PCR efficiency for all primer sets, dilution series of the PCR product encompassing both primer binding sites was used as template for real-time qPCR analysis. Plotting the Ct value against the logarithm of dilution factor should theoretically result in a linear curve with a slope of approximately -3.32 (=-^2^log(10)). In addition, this approach allowed us to determine the upper Ct limit, i.e. the highest Ct value that still was in the linear range. All primer sets showed an comparable amplification efficiency over a broad range of concentrations (Figure 1B and Table 3). These data confirmed that all primers have a comparable performance in qPCR.

**Table 2:** Primers targeting human integrins.

Target	Forward primer (5' - 3')	Reverse primer (5' - 3')
ITGα1	GGTTCCTACTTTGGCAGTATT	AACCTTGTCTGATTGAGAGCA
ITGα2	GGAACGGGACTTTCGCAT	GGTACTTCGGCTTTCTCATCA
ITGα3	AAGGGACCTTCAGGTGCA	TGTAGCCGGTGATTTACCAT
ITGα4	GCTTCTCAGATCTGCTCGTG	GTCACTTCCAACGAGGTTTG
ITGα5	TGCAGTGTGAGGCTGTGTACA	GTGGCCACCTGACGCTCT
ITGα6	TTGAATATACTGCTAACCCCG	TCGAAACTGAACTCTTGAGGATAG
ITGα7	CTGTTTCAGCTACATTGCAGTC	GCCTGGTGCTTGGGTTCT
ITGα11	GGAGGAAGACTTGCGTCG	CACAGGTTCCCCAGTAGATG
ITGαV	AATCTTCCAATTGAGGATATCAC	AAAACAGCCAGTAGCAACAAT
ITGβ1	GAAGGGTTGCCCTCCAGA	GCTTGAGCTTCTCTGCTGTT
ITGβ3	CCGTGACGAGATTGAGTCA	AGGATGGACTTTCCACTAGAA
ITGβ4	AGACGAGATGTTCAGGGACC	GGTCTCCTCTGTGATTTGGAA
ITGβ5	GGAGCCAGAGTGTGGAAACA	GAAACTTTGCAAACTCCCTC
ITGβ6	TCAGCGTGACTGTGAATATCC	GTGACATTTGGAGCTGTTCAC
ITGβ8	AATTTGGTAGTGGAAGCCTATC	GTCACGTTTCTGCATCCTTC
bACT	GCTGTGCTACGTCGCCCTG	GGAGGAGCTGGAAGCAGCC
cycloA	CTCGAATAAGTTTGACTTGTGTTT	CTAGGCATGGGAGGGAACA
b2MG	TCCATCCGACATTGAAGTTG	CGGCAGGCATACTCATCTT
HPRT1	AGAATGTCTTGATTGTGGAAGA	ACCTTGACCATCTTTGGATTA

ITG = integrin, bACT = beta-actin, cycloA = cyclophilin A, b2MG = beta-2-microglobulin, HPRT = hypoxanthine-guanine phosphoribosyltransferase 1.

**Table 3:** Characteristics of human integrin primer pairs in qPCR.

Integrin	fragment length (bp)	upper limit (Ct)	slope
**Alpha1**	129	26.1	-3.38
**Alpha2**	154	31.2	-3.04
**Alpha3**	129	29.0	-3.38
**Alpha4**	131	36.4	-3.41
**Alpha5**	88	32.0	-3.23
**Alpha6**	113	30.5	-3.26
**Alpha7**	150	31.9	-3.43
**Alpha11**	123	34.0	-3.36
**AlphaV**	140	33.9	-3.31
**Beta1**	107	34.3	-3.23
**Beta3**	132	30.1	-3.30
**Beta4**	115	29.8	-3.12
**Beta5**	143	34.4	-3.39
**Beta6**	155	32.6	-3.33
**Beta8**	146	33.6	-3.50

ITG = integrin, bp = base pairs, Ct = Cycle threshold.

### Integrin alpha5, beta1, and beta3 expression are prognostic factors for overall survival in early stage NSCLC patients

**Figure 2:**
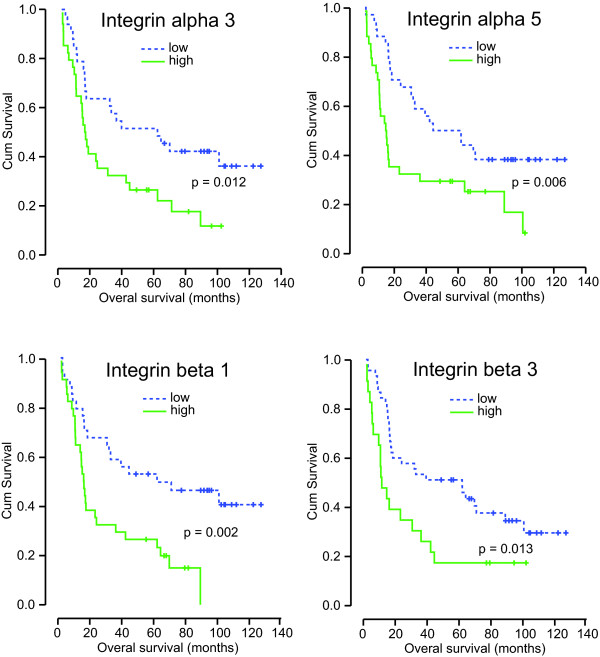
**Integrin expression profile and overall survival in NSCLC**. Kaplan Meier plot of overall survival (OS) of patients with detectable or undetectable integrin beta3 expression and of patients with low (below median) or high (above median) expression of the indicated integrins.

Next, the integrin expression profile of all the NSCLC tissues from the total patient group was determined. The expression levels of the different integrins varied considerably with integrin alphaV, beta1, beta4 and beta5 displaying the highest expression levels (Figure 1C). The expression of integrin alpha1, alpha2, alpha3, and alpha5 was lower and hardly any integrin alpha4 and integrin beta3 was detected. In addition, while most integrins were expressed at detectable levels, i.e. below the upper Ct limit, integrin alpha1 and integrin beta3 were only detectable in respectively 42% and 33% of the patients. Next, we analyzed the overall survival in patients grouped according to the expression level of a specific integrin. This revealed that patients which express integrin alpha3, alpha5, beta1, or beta3 above median levels have a significant shorter overall survival compared to patients whose integrin expression levels are below the median (Figure 2 and Table 4).

**Table 4:** Integrin expression and overall survival

Integrin	Median OS (95% CI) in months		
	Low expression*	High expression	P
**Alpha1**	39.3 (0.0-97.4)	23.2 (0.0-46.8)	Ns
**Alpha2**	31.9 (0.0-81.1)	18.4 (0.0-46.4)	Ns
**Alpha3**	61.8 (21.1-102.5)	16.2 (12.2-20.2)	**0.012**
**Alpha4**	17.5 (0.0-50.5)	30.6 (10.1-51.2)	Ns
**Alpha5**	44.4 (12.1-76.6)	14.7 (9.0-20.4)	**0.006**
**Alpha6**	31.9 (15.0-48.8)	36.1 (0.0-73.6)	Ns
**Alpha7**	31.9 (0.0-63.8)	23.2 (0.0-52.2)	Ns
**Alpha11**	31.9 (10.0-53.8)	32.8 (0.0-71.5)	Ns
**AlphaV**	23.2 (0.0-56.1)	30.6 (8.7-52.5)	Ns
**Beta1**	61.8 (0.0-129.3)	16.1 (13.2-19.0)	**0.002**
**Beta3**	61.8 (22.8-100.8)	11.5 (5.4-17.6)	**0.013**
**Beta4**	36.1 (20.6-51.5)	16.3 (14.1-18.5)	Ns
**Beta5**	24.0 (3.5-44.6)	23.2 (0.0-50.8)	Ns
**Beta6**	32.8 (4.5-61.1)	31.9 (0.0-64.5)	Ns
**Beta8**	23.2 (3.0-43.4)	36.1 (1.2-71.0)	Ns

*Low = below median, High = above median

### Integrin expression as marker for lymph node metastasis and recurrence

**Figure 3:**
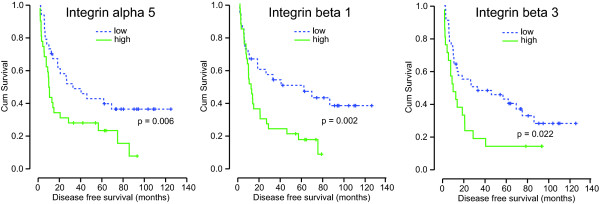
**Integrin expression and disease free survival in NSCLC**. Kaplan Meier plots of disease free survival (DFS) of patients with detectable or undetectable integrin beta3 expression and of patients with low (below median) or high (above median) expression of the indicated integrins.

Of the 68 patients included in this study, 19 patients (28%) had positive lymph nodes (N1) at the time of diagnosis. There was no difference in the integrin expression levels between patients with N1 disease and N0 disease **(data not shown)**. Apparently, integrin expression in the primary tumor does not predict the presence of lymph node metastasis. However, similar as for the overall survival, Kaplan Meier analysis indicated that elevated expression of integrin alpha5, beta1, or beta3 was associated with shorter disease free survival DFS (Figure 3 and Table 5).

**Table 5:** Integrin expression and disease free survival

Integrin	Median DFS (95% CI) in months		
	Low expression*	High expression	P
**Alpha1**	14.8 (0.0-64.2)	18.4 (8.3-28.5)	Ns
**Alpha2**	20.8 (0.0-46.5)	18.4 (9.1-27.7)	Ns
**Alpha3**	32.8 (0.0-73.3)	13.0 (8.5-17.4)	Ns
**Alpha4**	13.8 (0.0-29.4)	18.6 (2.6-34.6)	Ns
**Alpha5**	32.8 (5.7-59.9)	10.0 (8.4-11.6)	**0.018**
**Alpha6**	20.8 (10.1-31.5)	26.4 (2.2-50.6)	Ns
**Alpha7**	18.6 (1.0-36.5)	20.8 (0.0-42.0)	Ns
**Alpha11**	20.8 (3.4-38.2)	26.4 (0.0-58.5)	Ns
**AlphaV**	14.8 (0.0-37.1)	18.4 (8.6-28.2)	Ns
**Beta1**	61.8 (8.6-115.0)	12.4 (8.2-16.7)	**0.012**
**Beta3**	32.8 (0.0-79.4)	9.9 (2.8-17.1)	**0.022**
**Beta4**	20.9 (7.4-34.4)	13.0 (4.3-21.7)	Ns
**Beta5**	14.8 (2.0-27.7)	20.8 (0.0-46.1)	Ns
**Beta6**	26.4 (12.0-40.8)	14.8 (2.6-27.0)	Ns
**Beta8**	10.7 (0.0-25.0)	26.4 (4.2-48.6)	Ns

*Low = below median, High = above median

### Integrin expression in different histological subtypes of NSCLC

**Figure 4:**
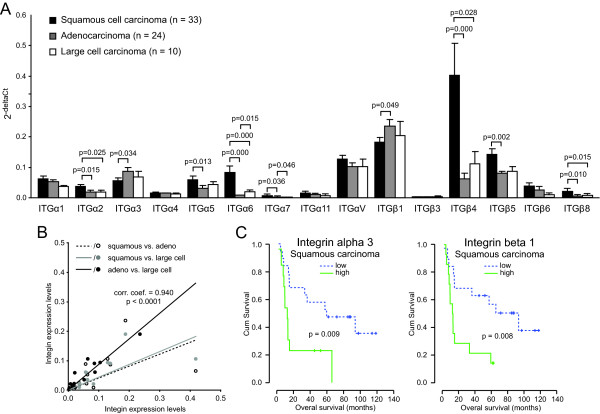
**Integrin expression profile and survival in subtypes of NSCLC**. **A) **Bar graph showing the subtype specific integrin expression. **B) **Scatter plot showing the correlation in integrin expression between the different subtypes of NSCLC. **C) **Kaplan Meier plots of overall survival of patients with low (below median) or high (above median) expression of the indicated integrins in squamous carcinoma.

Finally, the integrin expression of the 3 main histological subtypes included in this study, i.e. squamous cell carcinoma, adenocarcinoma, and large cell carcinoma, was compared. Integrin alpha2 and beta4 were significantly higher expressed in squamous cell carcinomas as compared to both adenocarcinoma and large cell carcinoma (Figure 4A). The same trend was observed for integrin alpha5 and integrin beta5, albeit only statistically significant for squamous carcinoma vs. adenocarcinoma. The latter also appeared to be characterized by increased expression of integrin alpha3 and beta1 as compared to squamous carcinoma. A summary of the fold-differences in integrin expression between the different histological subtypes is given in Table 6. Correlation analysis indicated that the integrin expression profile of squamous cell carcinomas appeared to differ most from both adenocarcinoma and large cell carcinoma. Indeed, the expression profiles of the latter two showed the strongest correlation with a correlation coefficient of 0.940 (p < 0.0001) (Figure 4B). Since the adenocarcinoma and large cell carcinoma cohorts were to small, the prognostic value for overall survival was only performed in patients with squamous cell carcinoma. Within this group, high expression of integrin alpha5 and beta1 were associated with shorter overall survival (Figure 4C).

**Table 6:** Fold expression differences between NSCLC subtypes.

Integrin	Adeno vs. Squamous	Large cell vs. Squamous	Large cell vs. Adenoma
**Alpha1**	0.8	0.6	0.7
**Alpha2**	0.5*	0.5*	1.0
**Alpha3**	1.5*	1.2	0.8
**Alpha4**	0.9	0.9	1.0
**Alpha5**	0.5*	0.8	1.4
**Alpha6**	0.1*	0.2*	2.4*
**Alpha7**	0.4*	0.3*	0.7
**Alpha11**	0.6	0.4	0.7
**AlphaV**	0.8	0.8	1.0
**Beta1**	1.3*	1.1	0.9
**Beta3**	0.7	1.0	1.5
**Beta4**	0.2*	0.3*	1.8
**Beta5**	0.6*	0.6	1.1
**Beta6**	0.6	0.3	0.4
**Beta8**	0.2*	0.4*	1.7

*statistically significant. For p-value see Figure 4.

## Discussion

In order to select patients with a high risk of recurrence, mRNA expression of multiple integrins by qPCR in patients with resectable NSCLC was determined. To our knowledge, this is the first study that assessed the expression of such a large panel of integrins simultaneously in NSCLC patients. Only recently, Guo et al. performed extensive integrin expression profiling in different lung cancer cell lines[16]. Unfortunately, they did not address integrin expression in tumor samples from NSCLC patients. Nevertheless, they also identified integrin beta1 as the most abundantly expressed integrin while integrin alpha4 was hardly detectable. This is in agreement with our observations. On the other hand, all the cell lines expressed integrin beta3 which was only detected in a subset of our patients. The observed differences are most likely related to the different cellular compositions and environmental conditions in tumor tissues compared to cultured tumor cell lines. Despite these differences, the overall expression signatures were largely comparable which is in agreement with earlier findings[17].

An interesting observation in the current study was that increased expression of integrin alpha5 and integrin beta1 predicts shorter overall survival and shorter disease free survival of NSCLC patients. An association between increased integrin alpha5 or beta1 expression and poor patient outcome in NSCLC has been reported previously[8,11,18]. For example, Okamura et al. found that increased integrin beta1 expression was a prognostic factor for poor overall survival in NSCLC patients[18]. Adachi et al. reported a significant worse 5-year survival of node-negative NSCLC patients with high integrin alpha5 expression. It was suggested that these patients might have had undetected micro-metastases at time of surgery or that they were more prone to metastasis[8]. Interestingly, Han *et al*. found that increased expression of integrin alpha5 as well as integrin beta1 was associated with lymph node metastasis in NSCLC patients[11]. We could not find an association between the expression of both integrins and lymph node metastasis. On the other hand, we did observe that elevated expression of both integrin alpha5 and integrin beta1 were prognostic factors for recurrence. All these data clearly show that both integrins are associated with a more aggressive phenotype in NSCLC, although the underlying mechanisms remains to be elucidated. Possibly, elevated expression of integrin alpha5beta1 increases the invasive potential of lung tumor cells as shown by Takenaka et al. using the human lung adenocarcinoma cell line PC9[19]. They found that a highly metastatic variant of PC9 cells had increased integrin alpha5beta1 expression and that the metastatic potential could be reduced more than half by treatment with an anti-beta1 antibody[19].

Apart from integrin alpha5 and beta1, it was also observed that increased integrin beta3 expression was as prognostic factor for overall and disease free survival. However, integrin beta3 expression was only detected in approximately one-third of the patients. Integrin beta3 has been shown to be exclusively expressed by the endothelium of large vessels of normal lung tissue and not in other tissue types of the normal lung[20]. Thus, the absence of integrin beta3 may reflect a down-regulation of this integrin or indicate an impaired vascularization of a subset of tumors. Furthermore, integrin beta3 is often found to associate with integrin alphaV in activated endothelial cells during tumor angiogenesis. So, the elevated integrin beta3 expression in patients with worse prognosis might indicate increased angiogenesis activity. However, we did not find correlation between the expression of integrin alphaV and beta3 nor did integrin alphaV expression associate with patient survival, lymph node metastasis or recurrence. In fact, we observed relative high expression levels of integrin alphaV which is contradiction with Smythe *et al*. who found that loss of integrin alphaV is associated with a high risk for recurrence[12]. This discrepancy could be explained by the fact that the latter study assessed protein expression while we determined mRNA expression levels. Studies to determine the exact relation between integrin expression and the angiogenic activity are ongoing.

Another interesting observation was that the different subtypes of NSCLC have distinct integrin expression profiles. We observed higher expression of integrin alpha2 and beta4 specifically in squamous cell carcinoma which is in agreement with a previous study by Koukoulis et al. [21]. Furthermore, using an immunohistochemical approach they were unable to detect integrin alpha4 and integrin beta3 protein expression which also corroborates with the low mRNA levels detected in this study. On the other hand, they could not detect integrin alpha4 or alpha5 in any of the subtypes while the latter was identified as a prognostic factor in the current study. This is most likely related to the experimental approach since another study that analyzed integrin mRNA levels also reported alpa4 and alpha5 expression[16]. In fact, the differences in expression that we observed between squamous and adenocarcinomas were largely in agreement with the differences found when adenocarcinoma cell line A549 was compared to squamous cell line Calu-1 in that same study[16]. Recently, Boelens *et al*. reported on increased expression of integrin alpha3 in adenocarcinoma compared to squamous cell carcinoma which is in accordance with our results[10]. They also found elevated integrin beta4 expression in squamous cell carcinoma as compared to adenocarcinoma[10], similar as in our study. Altogether, these studies indicate that integrin expression profiles in adenocarcinoma and large cell carcinoma profiles are most alike and most different from squamous cell carcinoma. The latter is the most common histological type, it is central in location and most often late in development of distant metastases. Interestingly, adenocarcinoma is characterized by the early development of metastases[22]. Since integrins are involved in many processes associated with metastasis like loss of intracellular adhesion within the primary tumor, tumor cell entry into the lymphatic or blood vessels, and adherence of the tumor cells the endothelium at distant sites[23], the observed differences in integrin expression between both subtypes might underlie the different metastatic potential. Furthermore, while this study focused on NSCLC, integrins are of course implicated in tumor progression and metastasis of many epithelial derived tumors[24-26]. For future studies it might be worthwhile to compare integrin expression profiles in different epithelial tumors to identify common themes in epithelial tumor progression.

In summary, this is the first study that correlates extensive integrin mRNA expression profiling to the prognosis of early stage NSCLC patients. Our findings identified integrin alpha5, beta1, and beta3 as prognostic markers for overall survival and disease free survival. In addition we found distinct integrin expression profiles that can differentiate between adenocarcinoma and squamous cell carcinoma of the lung. These findings indicate that changes in integrin expression in NSCLC play an important role in the development and behavior of NSCLC which influence the survival of the patient. Determining the integrin expression profile might serve as a tool in predicting the prognosis of individual patient and selection of patients who have a high risk of recurrence and will benefit from adjuvant treatment.

## Competing interests

The authors declare that they have no competing interests.

## Authors' contributions

VvdB, BAV and VT carried out the experiments. VvdB and BAV collected the patient data and helped to draft the manuscript. R-JvS evaluated the tissue sections. AWG participated in the design of the study. AMD and VT conceived of the study, and participated in its design and coordination and wrote the manuscript. All authors read and approved the final manuscript.
